# Clinical Manifest X-Linked Recessive Adrenoleukodystrophy in a Female

**DOI:** 10.1155/2013/491790

**Published:** 2013-06-23

**Authors:** Gyda Hlin Skuladottir Jack, Karolina Malm-Willadsen, Anja Frederiksen, Dorte Glintborg, Marianne Andersen

**Affiliations:** ^1^Department of Endocrinology, Odense University Hospital, Kløvervænget 6, 3rd Floor, 5000 Odense C, Denmark; ^2^Department of Clinical Genetics, Vejle Hospital, Kabbeltoft 25, 7100 Vejle, Denmark

## Abstract

Adrenoleukodystrophy (ALD) is a rare X-linked inherited leukodystrophy with a reduced capacity for degradation of very long chain fatty acids (VLCFAs). The intracellular accumulation of VLCFA leads to demyelination in the central nervous system (CNS) and cell destruction in the adrenal glands. ALD primarily affects males; however, females may develop milder symptoms that may be difficult to recognize. The present report describes a 35-year-old female who experienced a feeling of heaviness in the upper and lower limbs, pain in both knees, and difficulty climbing stairs, running, and jumping. Clinical examination revealed decreased sensitivity in the feet, particularly to touch. Deep tendon reflexes in the lower limbs were brisk, and Babinski's sign was present bilaterally. Multiple sclerosis (MS) was excluded, and all clinical and biochemical tests were normal. After two years of progressing symptoms, the patient was reevaluated and plasma levels of VLCFA were found to be elevated. Seven years prior to this finding, the patient had been found to be heterozygous for the missense mutation c.1679C> T, p.Pro560Leu on the *ABCD1* gene (ATP-Binding Cassette subfamily D1). In conclusion, the patient's symptoms could be attributed to ALD. The present case underlines the importance of reevaluating family history in women presenting with vague neurological symptoms.

## 1. Introduction

Adrenoleukodystrophy (ALD) is an X-linked inherited condition, primarily affecting the central nervous system (CNS) and the adrenal glands [1]. The symptoms vary from mild impaired vibration sensation in lower extremities to progressive paralysis in all four limbs. Symptoms may also include adrenal insufficiency [2]. The present case presents a female with vague neurological symptoms attributed to ALD. 

## 2. Method

The present patient has given her informed consent to participate in this research project. Therefore, it is not relevant to get approval from The National Danish Committee on Health Research Ethics (Videnskabsetisk Komité, VEK).

## 3. Case Report

A 35-year-old Caucasian, right-handed female had for 1.5 months experienced a feeling of heaviness in the upper and lower limbs, pain in both knees, and difficulty climbing stairs, running, and jumping. The patient also had balance problems. She worked in a nursing home, but due to her symptoms she was only able to work part time. Clinical examination revealed normal muscle strength and normal muscle tonus in the arms and legs. There were no muscular atrophy and normal gait. There was decreased sensitivity in the feet, particularly to touch. Deep tendon reflexes in the lower limbs were brisk, and Babinski's sign was present bilaterally. There were no cranial nerve symptoms. All paraclinical examination was normal, including magnetic resonance imaging (MRI) of the cerebrum and corticospinal tract, cerebrospinal fluid, visual evoked potentials, and somatosensory evoked potentials. There was no apparent explanation for the patient's symptoms, and the investigation was considered completed. 

**Figure 1:**
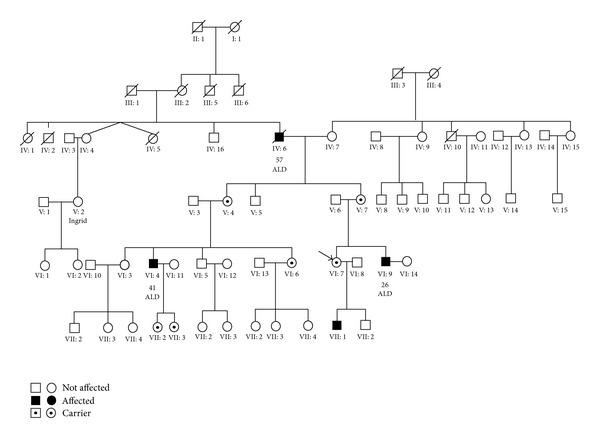
Genetic family diagram. The affected 35-year-old female is marked.

After two years of progressing symptoms, the patient was reevaluated. The patient's plasma levels of VLCFA were measured and found to be elevated; C22 : 0 = 28 *μ*mol (normal range 18–60 *μ*mol), C24 : 0 = 44 *μ*mol (normal range 16–50 *μ*mol), C26 : 0 = 1.2 *μ*mol (normal range 0.1 to 0.7 *μ*mol), C24 : 0/C22 : 0 ratio = 147 (normal range 40–115), and C26 : 0/C22 : 0 ratio = 6.8 (normal range 0–3). Seven years prior to this finding, the patient had been found to be heterozygous for the missense mutation c.1679C> T, p.Pro560Leu on the *ABCD1* gene (ATP-Binding Cassette subfamily D1). She had been screened as part of a genetic family investigation, since her brother had adrenal insufficiency and was positive for this mutation. The patient's mother and aunt were found to be asymptomatic carriers of the mutation. The patient's son, her male cousin, and her maternal grandfather were found to be mutation positive and had symptoms of ALD (see Figure 1). The family history included a case of MS, as the patient's father had been diagnosed with the disease in a young age. 

It was concluded that the patient's symptoms could be attributed to the fact that she carried the ALD mutation. Adrenocorticotropic hormone (ACTH) levels were normal, indicating that she did not have adrenal insufficiency. The patient was treated with Lorenzo's oil, physiotherapy, and low fat diet.

## 4. Discussion

ALD is an X-linked inherited condition, primarily affecting males (incidence 1 : 16.800) [3], which presents with two main phenotypes. The most common form, the cerebral inflammatory phenotype, affects boys 3–10 years of age [1]. They present with progressive behavioural disorder, learning disabilities, impaired vision, poor hearing, and progressive paralysis in all four limbs. This form of ALD often leads to total disability within 3 years and death at a young age [3]. The other main phenotype, adrenomyeloneuropathy (AMN), is a milder form of ALD, with later onset and slower progression. AMN affects the ascending and descending tracts of the spinal cord [1]. Approximately half of ALD mutation positive females present with mild or moderate neurological symptoms including slowly progressive paraparesis, impaired vibration sensation in lower extremities, and objective findings such as hyperreflexia and Babinski's sign. Symptoms may also include bowel and bladder difficulties [4, 5]. The present patient experienced some of these AMN-like symptoms when she first sought medical attention, and she is likely to have the AMN subtype of ALD. The two phenotypes can be differentiated clinically, not biochemically. 

The epigenetic phenomenon of X-inactivation may contribute to the variable clinical presentations of X-linked conditions, such as ALD [5]. In the female embryo, there is a dosage reduction of the majority of the genes at one of the two X-chromosomes to equal the dosage in males. A random and irreversible process inactivates either the maternal or the paternal inherited X-chromosome. Normally the distribution is around the mean (50 : 50), but in rare cases there is an unequal distribution (>80 : 20) in favour of the X-chromosome harbouring the mutation allele. The phenomenon skewed X-inactivation can lead to clinical manifestations of X-linked diseases in females [5–7]. ALD females commonly present with milder neurological symptoms, and while adrenal insufficiency is common in men with ALD, only 1% of the mutation positive females develop this condition [1, 3]. Due to the risk of developing adrenal insufficiency, annual ACTH tests can be conducted to monitor adrenal function. The present patient had a normal ACTH test and is annually monitored. 

Males with ALD are born with elevated plasma levels of VLCFA. The plasma levels of VLCFA can therefore be used for diagnostic purposes. Most mutation positive females have elevated plasma levels of VLCFA, but up to 20% of affected female patients have normal VLCFA levels. Therefore, measurement of VLCFA cannot be used for reliable diagnosis. Instead, detection of mutation in the ABCD1 gene verifies the diagnosis genetically [8]. The present patient's symptoms could have been attributed to her ALD mutation carrier status upon the debut of her symptoms. 

The treatment possibilities in ALD are limited. Lorenzo's oil (LO) is a 4 : 1 mixture of glyceryl trioleate and glyceryl trierucate, which may stabilize VLCFA plasma levels when combined with a low fat diet [1]. A single arm study of 45 AMN men suggested that progression of neurological symptoms during LO therapy was significantly slower than during the pretreatment period [2]. However, no randomized controlled trials (RCTs) have been conducted to confirm that LO can stop the progression of neurological symptoms in ALD patients. Additionally, physical therapy may reduce the severity of the symptoms. Adrenal insufficiency is treated with glucocorticoid and mineralocorticoid replacement [9]. Alternative treatment for boys with the cerebral inflammatory phenotype consists of hematopoietic stem cell transplantation [10]. Attempts with immune therapy have not been successful [2]. 

In conclusion, ALD in females is difficult to diagnose due to its vague symptoms. ALD overlaps three medical specialties (neurology, clinical genetics, and endocrinology) and is rare, and the symptoms include slowly progressing stiffness and weakness of legs, impaired vibration sensation, urinary sphincter disturbances, and adrenal insufficiency. In the present case, the family history was the key information to a correct diagnosis. Attention can be drawn to the connection between an ALD family history and middle-aged females presenting with vague neurological symptoms.
